# High-Performance SAW Resonator with Spurious Mode Suppression Using Hexagonal Weighted Electrode Structure

**DOI:** 10.3390/s23249895

**Published:** 2023-12-18

**Authors:** Yulong Liu, Hongliang Wang, Feng Zhang, Luhao Gou, Shengkuo Zhang, Gang Cao, Pengcheng Zhang

**Affiliations:** National Key Laboratory for Electronic Measurement Technology, Key Laboratory of Instrumentation Science and Dynamic Measurement, Ministry of Education, North University of China, Taiyuan 030051, China; sz202106081@st.nuc.edu.cn (Y.L.); b20210629@st.nuc.edu.cn (F.Z.); s202106043@st.nuc.edu.cn (L.G.); sz202106120@st.nuc.edu.cn (S.Z.); s202106099@st.nuc.edu.cn (G.C.); s202106145@st.nuc.edu.cn (P.Z.)

**Keywords:** surface acoustic wave (SAW), hexagonal weighted resonator, high Q value, suppress spurious modes, MEMS process

## Abstract

Surface acoustic wave resonators are widely applied in electronics, communication, and other engineering fields. However, the spurious modes generally present in resonators can cause deterioration in device performance. Therefore, this paper proposes a hexagonal weighted structure to suppress them. With the construction of a finite element resonator model, the parameters of the interdigital transducer (IDT) and the area of the dummy finger weighting are determined. The spurious waves are confined within the dummy finger area, whereas the main mode is less affected by this structure. To verify the suppression effect of the simulation, resonators with conventional and hexagonal weighted structures are fabricated using the micro-electromechanical systems (MEMS) process. After the S-parameter test of the prepared resonators, the hexagonal weighted resonators achieve a high level of spurious mode suppression. Their properties are superior to those of the conventional structure, with a higher Q value (10,406), a higher minimum return loss (25.7 dB), and a lower ratio of peak sidelobe (19%). This work provides a feasible solution for the design of SAW resonators to suppress spurious modes.

## 1. Introduction

With the invention of the interdigital transducer (IDT), surface acoustic wave (SAW) technology has developed rapidly because the IDT can effectively excite and detect surface acoustic waves on piezoelectric substrates. As one of the most common SAW devices, SAW resonators are widely used in both communication [1] and sensing [2] applications, such as filters and sensors.

SAW resonators are an important part of SAW sensors and can be fabricated with different substrate materials to measure various physical quantities. For instance, a substrate combining magnetic and piezoelectric materials can be made into a magnetic field sensor [3]. The substrate of the temperature sensor is a temperature-sensitive piezoelectric material LGS [4]. Furthermore, to measure the strain, a thinner substrate is used under the IDT to fabricate strain sensors [5], etc. As for filters, SAW filters can be formed by connecting multiple resonators in series and parallel. [6]. In addition, Chao et al. used a high quality factor quartz tuning fork [7]. All of these devices require resonators that have a high quality factor, high minimum return loss, and effective suppression of spurious modes. At present, many scholars’ research focuses on how to improve the performance of SAW resonators. The transverse spurious mode produced by conventional electrode configurations consumes a small amount of power from the wireless radio frequency (RF) interrogation signal, resulting in a decrease in the signal-to-noise ratio. On the other hand, it will also increase the system’s wireless signal misinterpretation rate, complicate subsequent demodulation, and deteriorate filtering effectiveness [8].

To suppress spurious waves, several methods have been explored. The first method for suppressing spurious waves is called the piston-mode technique, which involves shaping the mode to match the rectangular shape of the excitation. This technique was first proposed by Kaitila et al. [9] for bulk acoustic wave (BAW) resonators. This method demands very high precision in the fabrication process, making it challenging to strictly control the shape of the interdigital transducer (IDT). The left-off process used in this paper is challenging to fabricate. The second method, involving a tilted transducer, may lead to unnecessary waste of acoustic energy [10]. Another method focuses on matching the displacement shape of the main resonance to the dummy finger shape by weighting the IDT in the transverse direction. This technique was first proposed by Shimizu et al. [11], and was first used to achieve a small Love wave resonator with a very small capacitance ratio without a reflector. Meanwhile, the authors used a smaller number of IDT pairs to obtain a larger bandwidth in filter applications. Compared with the conventional apodization method for spurious mode suppression, although this approach does not seriously reduce the effective aperture of the resonator, the effective aperture is also lost to a certain extent. In addition, the smaller number of IDT pairs cannot guarantee excitation strength, limiting its suitability for SAW devices. To address the shortcomings of the above method, this paper improves the rhombus-weighted structure to obtain a high-performance resonator suitable for SAW devices.

In this work, the technique of hexagonal weighting is proposed to realize the high-performance SAW resonator mentioned above. Firstly, finite element simulation is conducted on an X-112°Y-cut LiTaO_3_ substrate. The analysis reveals that the frequency values of the Rayleigh wave and shear-horizontal wave under the cut angle are quite different. This result largely avoids the coupling of the two modes, making it easier to investigate the spurious mode suppression of the Rayleigh wave separately. The SAW resonator designed in this paper uses Al as the electrode material, and the thickness, electrode pairs, and aperture width of the IDT are analyzed using the finite element method. A solution of a hexagonal weighted IDT is proposed for the spurious modes existing in the conventional structure. This solution uses regular and variable electrode lengths to suppress spurious modes of each order as much as possible. Theoretical verification and simulation confirm that the fifty percent weighting scheme results in higher Q values and minimum return loss. Eventually, two resonators are fabricated using the MEMS process to verify the theoretical and simulation results. Thus, the weighted resonators designed in this work have great prospects for future applications in the field of SAW devices.

## 2. Theory and Simulation

### 2.1. Theoretical Model

In this paper, the performance of the hexagonal weighted SAW resonator is simulated and analyzed using COMSOL Multiphysics 6.0 software. Surface acoustic wave (SAW) devices, during their operation, are governed by differential equations that need to be solved, taking into account certain parameters: geometric complexity of the device, properties of the material, and boundary conditions [12]. The finite element method (FEM) provides numerical solutions defined by associated differential formulas. The formulas that make the link between stress, deformation, electric field, and electric displacement field in the stress charge of a piezoelectric crystal are given by [13]:

(1) $$T_{ij}=C_{ijkl}^{E}S_{kl}-e_{ijk}E_{k}$$

(2) $$D_{i}=e_{jkl}S_{kl}+\varepsilon_{ij}^{s}E_{j}$$

where $T_{ij}$ represents the stress vector; $C_{ijkl}$ represents the elasticity matrix (N/m^2^); $e_{ijk}$ represents the piezoelectric matrix (C/m^2^); $\varepsilon_{ij}$ represents the permittivity matrix (F/m); $E_{k}$ represents the electric field vector (V/m); $S_{kl}$ represents the strain vector; and $D_{i}$ represents the electrical displacement (C/m^2^). The degrees of freedom are the displacements in the global x, y, and z directions. The potential (V) can be derived by solving Newton’s and Maxwell’s formulas associated with (1) and (2) [14]:

(3) $$\sum_{ijk}C_{ijkl}^{E}\;\frac{\partial^{2}u_{l}}{\partial x_{j}\partial x_{k}}+\sum_{jk}e_{kij}\;\frac{\partial^{2}u_{l}}{\partial x_{j}\partial x_{k}}=\rho \frac{\partial^{2}u_{i}}{\partial t^{2}}$$

(4) $$\sum_{kl}e_{jkl}\;\frac{\partial^{2}u_{l}}{\partial x_{j}\partial x_{k}}-\sum_{jk}\varepsilon_{jk}^{S}\frac{\partial^{2}\varphi }{\partial x_{j}\partial x_{k}}=0$$

i, j, k and l in the Equations (1)–(3).

Here, $u_{i}$ represents the component of the elastic displacement; u represents the potential of the wave; $C_{ijkl}$, $e_{ikl}$, and $e_{kij}$ are the components of the elastic, piezoelectric, and dielectric constant tensor, respectively; and ρ represents the mass density. For thinner piezoelectric substrates, the stress distribution in the plane can be estimated with the above formulas.

The energy of SAW is affected by losses that include mechanical, dielectric, and piezoelectric losses. For anisotropic materials, we can introduce the viscosity factor $\eta_{C}^{m,n}$, dielectric loss factor $\eta_{\varepsilon }^{m,n}$, and piezoelectric loss factor $\eta_{e}^{m,n}$ to consider these losses for the device, and, in this case, $C_{ijkl}^{m,n}$, $\varepsilon_{ij}^{m,n}$, and $e_{ijk}^{m,n}$ are shown in plural forms [15,16,17].

(5) $${\bar{C}}_{ijkl}^{m,n}=\left(1+j\eta_{C}^{m,n}\right)C_{ijkl}^{m,n}$$

(6) $${\bar{\varepsilon }}_{ij}^{m,n}=\left(1-j\eta_{\varepsilon }^{m,n}\right)\varepsilon_{ijk}^{m,n}$$

(7) $${\bar{e}}_{ijk}^{m,n}=\left(1-j\eta_{e}^{m,n}\right)e_{ijk}^{m,n}$$

where i, j, k and l represent the components of each tensor.

In summary, the above formulas can provide theoretical support for the finite element model of the piezoelectric structure.

### 2.2. Resonator Parameters Design and Simulation

The most common type of surface acoustic wave resonator uses the Rayleigh wave, in which the coupling of the longitudinal shear waves limits the acoustic energy near the substrate surface, resulting in an out-of-plane elliptically polarized surface wave. The frequency (f) of such a wave is calculated by the following formula:

(8) $$f=\frac{v}{l}$$

where v represents the speed of sound for the substrate material and l represents the wavelength. As described in the perturbation theory [18], increasing the thickness of the metal that constitutes the IDT will affect the results, while the frequency can also be controlled by changing the geometry of the IDT, such as the metallization rate. A thicker metal film and larger metallization rate will increase the mass-loading effect and thus decrease the resonant frequency. Therefore, the simulation is necessary for accurately designing and manufacturing SAW devices, which can improve the resonator performance and shorten the device design cycle. The simulation model in this paper is established based on the optimization objectives.

**Figure 1:**
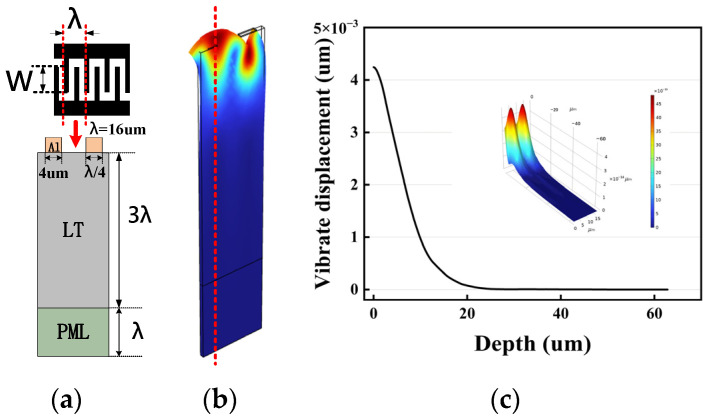
(**a**) Schematic diagram of the resonator slice model. (**b**) Simulation results of the SAW resonator in Rayleigh mode. (**c**) Displacement of the model in Rayleigh wave mode versus substrate depth.

In this study, the electrode finger width of the single-port resonator was set to 4 μm, the wavelength was 16 μm, and the expected frequency was approximately 210 MHz, which is a common frequency in SAW sensors [19]. The COMSOL Multiphysics model used in this study was built in a 3D structural mechanics module. Based on the study in reference [20], the metal ratio (a/p) was determined to be 50%. The gap length between the IDT and the reflectors was set to the width of one finger by default. Also, since the electrode fingers of the IDT were periodically distributed, a 3D cell-slicing finite element model was established, as shown in Figure 1a, which was used to guide the design of the resonator parameters.

Considering that the vibrations of the surface acoustic wave were mainly concentrated near the one wavelength, the substrate thickness was determined to be three times larger than the wavelength. In addition, in the perfect match layer (PML), a thickness of one wavelength was added at the bottom of the model to absorb the reflection of other acoustic waves. Meanwhile, to simulate an infinite IDT structure, periodic boundary conditions were applied around the periphery of the model. The simulation results are shown in Figure 1b. The material parameters used in this paper were obtained from reference [21]. The longitudinal displacement versus substrate depth is shown in Figure 1c, and the results clearly show that the vibration intensity decreased exponentially as the depth of the substrate increased. It can be seen that most of the vibration energy during Rayleigh wave propagation on the substrate was within one wavelength, which can also prove the reasonableness of the modeling. This is consistent with the research in reference [22].

**Figure 2:**
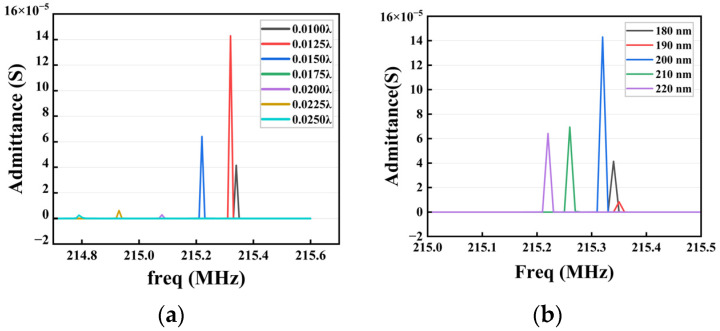
Admittance curves for different electrode thicknesses. (**a**) Electrode thickness from 0.010 λ to 0.025 λ. (**b**) Electrode thickness from 180 nm to 220 nm.

The phenomenon of electrode mass loading was introduced when the metal film covered the piezoelectric material, and the thickness variation of the electrode would directly affect the admittance of the SAW resonator [23]. When the admittance reached its maximum value, the impedance and losses of the designed surface acoustic wave sensor reached their minimum; thereby, the amplitude of the Rayleigh wave reached its maximum. Finally, the performance of the surface acoustic wave resonator was tested. Therefore, the relationship between electrode thickness and resonator admittance was analyzed using parametric sweeping. First, the electrode thickness varied between 0.010 λ and 0.025 λ in steps of 0.0025 λ, and the center frequency varied between 214.5 MHz and 215.4 MHz. The parametric simulation results are plotted in Figure 2a, which shows that the admittance reached relatively high levels when the thickness of the electrode was near 0.0125 λ. With this thickness, a more precise sweep was performed in the interval from 180 nm to 220 nm, with a step of 10 nm. As shown in Figure 2b, the value of the admittance was the maximum when the electrode thickness was 200 nm; the results of both simulations are consistent.

**Figure 3:**
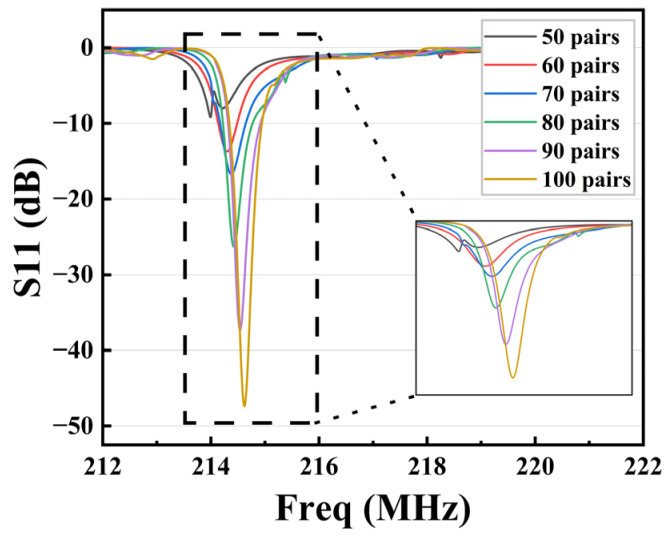
S11 parameter curves for different electrode pairs of IDTs.

When the device was resonant, the piezoelectric substrate and IDT parameters could be equated to the RLC circuit, whose static capacitance $C_{0}$ is given by Formula (9):

(9) $$C_{0}=N_{p}\left(\varepsilon_{r}\varepsilon_{0}+\varepsilon_{0}\right)W$$

where $C_{0}$ represents the static capacitance; $N_{p}$ represents the pair of electrodes; W represents the acoustic aperture width; $\varepsilon_{0}$ represents the dielectric constant under vacuum; and $\varepsilon_{r}$ represents the relative dielectric constant of the piezoelectric state. According to Formula (9), except for $\varepsilon_{0}$ and $\varepsilon_{r}$, which are self-relevant material parameters, both the $N_{p}$ and W of the resonator will cause an increase in static capacitance. This results in stronger coupling, which helps to reduce the return loss and increase the Q value of the resonators. The S11 parameter is expressed as the proportion of the reflected signal in the incident signal of the device, and the S11 value in logarithmic form is negative in this article, so the smaller the S11 value, the less the signal is lost within the device. Meanwhile, the S11 curve is steeper, which means the SAW resonator has a higher Q value. The following simulation results use this parameter to evaluate the performance of the device. After setting the electrode thickness of the 3D-sliced model to 200 nm, the model was simulated using a COMSOL array, resulting in simulation graphs for various pairs of electrodes. As shown in Figure 3, the S11 curves were compared to 50, 60, 70, 80, 90, and 100 pairs of electrodes. The chart shows that the sidelobe peak of the S11 curves was suppressed when the number of pairs of IDTs changed from 50 to 60 pairs, and the main resonant peak became more pronounced. Next, the main resonant peak increased sharply during the variation from 60 to 80 pairs of electrodes, but another spurious wave appeared on the right side of the main resonant peak at 80 pairs. When the number of electrode pairs was 90 and 100, there were no additional spurious waves, and the S11 value was lower and the main resonance peak was steeper. Additionally, the number of IDT pairs was inversely proportional to the bandwidth of the resonator, which means that a smaller bandwidth is beneficial for increasing the Q value of the resonator. Considering the performance and size of the overall device, 90 pairs of electrodes were chosen in this paper.

**Figure 4:**
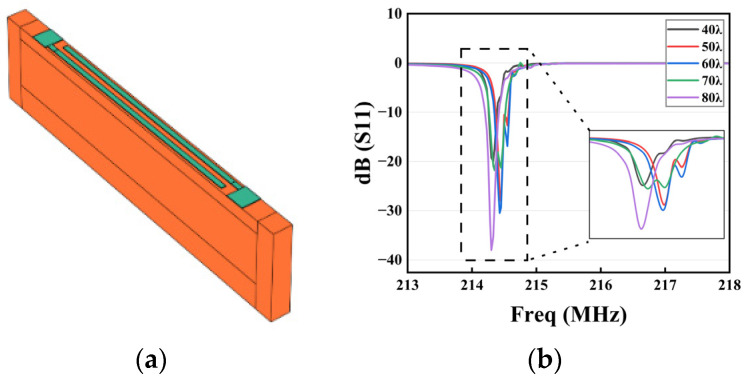
(**a**) Periodic strip 3D finite element model. (**b**) S11 parameter profiles of IDT with different W.

To simulate the coupled clutter waves of the shear-horizontal component of the LiTaO_3_ substrate, the IDT aperture width parameter W (Formula (9)) was added to the original model, which became a periodic strip 3D finite element model, and PML was also placed at both ends and the bottom to avoid boundary reflections. The strip 3D geometry consisting of the aperture, gap, and bus-bar regions in the transverse direction is shown in Figure 4a. To optimize the computational resources, periodic boundary conditions were applied on both sides of the elongated structure to simulate an infinite IDT. Additionally, grid refinement was exclusively implemented in the vicinity of the electrodes.

Figure 4b shows the S11 curves at different aperture widths. As the width of the aperture increased, it can be seen that the clutter gradually decreased, and the main resonance peak gradually increased from 18 dB at 40 λ to 38 dB at 80 λ. Both the main resonance and sidelobe peak were also increasing when the aperture width varied between 40 λ and 60 λ. When the aperture width was 70 λ, the peak of the sidelobe increased further, which directly affected the amplitude of the main resonance. Finally, when the aperture width increased to 80 λ, the peak of the resonance was suppressed to the maximum extent, and the resonance peak also became steeper, so the best aperture width was 80 λ. In short, either adding $N_{p}$ or W greatly improved the performance of the SAW resonator, but at the same time, the increases in $N_{p}$ and W were also accompanied by an increase in the device size, additional clutter, etc., so the choice of the two should be comprehensively considered.

According to the above simulation results, the structure parameters of the single electrode unweighted resonator are designed to obtain a lower S11 value. The detailed parameters are shown in Table 1.

The above simulations allowed us to find the best parameters to maximize the performance of the fabricated resonator. However, the periodic strip 3D finite element model was used to simulate an infinite IDT through the cell structure, which was different from the actual one. Not only that, in the completed device, the spurious modes would only be more severe and affect the resonator performance significantly. Therefore, after the basic parameters of the conventional resonator were determined, a weighted resonator structure was introduced to suppress the spurious waves.

### 2.3. Weighted IDT Resonator with Spurious Transverse Mode Suppression

**Figure 5:**
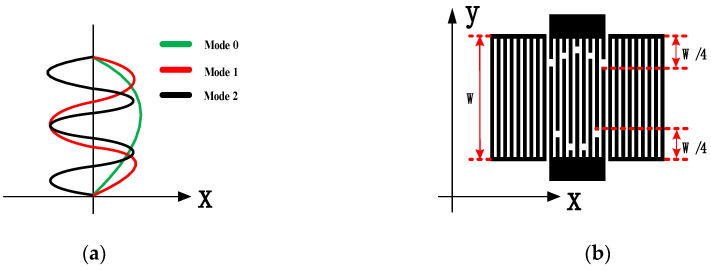
(**a**) Relative amplitude displacement contour curves for unweighted modes 0, 1, and 2. (**b**) Sketch of the single-port hexagonal weighted resonator.

When the surface acoustic wave propagates over the same length of the effective aperture, the spurious waves of each order can also be excited along with the main mode to affect M0. The spurious responses, which are caused by the anharmonic resonance of the transverse and longitudinal modes, can be eliminated by weighting the cross length [11]. In the IDT, the standing wave amplitude distribution in the longitudinal (propagating) direction varies co-sinusoidally. Figure 5a shows the vibration displacement curves for M0 and the spurious waves (M1 and M2). The spurious responses in such a two-dimensional amplitude distribution can be eliminated by weighting the cross length, where M0 of the weighted resonator is excited only by the fork finger electrode region, and other spurious modes penetrate into the dummy electrode region. These spurious modes are influenced more by the dummy electrode, whose scalar potential will eventually converge to zero, minimizing the effect on the resonator. Based on the above principle of weighting, in references [24,25], the authors simply reduced the loss of effective aperture size and fabricated the devices more simply. Though a weighted resonator in the above method can suppress spurious waves, the degree of weighting needs to be considered comprehensively; otherwise, other spurious waves will be introduced. In this study, the degree of weighting was characterized by the length of the weighted aperture as a percentage of the effective aperture length, as shown in Figure 5b, which is a simplified schematic of a hexagonal weighted resonator (with a weighting degree of 50%).

**Figure 6:**
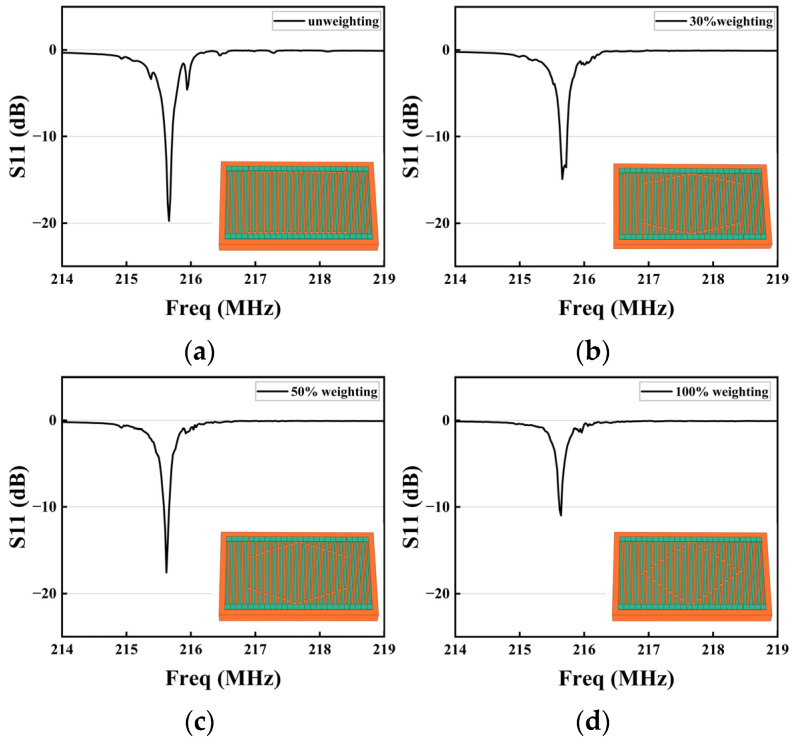
S11 curves of resonators with different weighting levels, the inset shows the simulation model. (**a**) Weighted at 0%. (**b**) Weighted at 30%. (**c**) Weighted at 50%. (**d**) Weighted at 100%.

In this study, simplified models of eighteen electrode pairs of IDTs and four electrode pairs of reflective grids were developed regarding the base parameters in Table 1. To determine a better weighting structure, four resonator models with different degrees of weighting were compared, as shown in the insets of Figure 6a–d. The four models were unweighted (conventional structure), weighted at 30%, hexagonal weighted (weighted at 50%), and rhombic weighted (weighted at 100%). By analyzing the simulation results in Figure 6, the resonator with a weighting degree of 30% again introduces new spurious waves that interfere with the main resonant peak. The resonators with hexagonal weighted and rhombic weighted structures had better suppression on sidelobe peaks of the S11 curve. However, compared to the rhombic weighted structure, the hexagonal weighted structure had a better resonance strength. This is because the former suppresses spurious modes while losing more effective aperture, resulting in a decrease in the strength of the electroacoustic excitation, and an increased value of S11.

### 2.4. Manufacturing Process and Morphological Characterization of SAW-Weighted Resonators

To verify the above simulation results, two types of resonators, a conventional resonator and a hexagonal weighted resonator were prepared, which were denoted as “Chip A” and “Chip B”, respectively. Due to the strong pyroelectric effect of lithium tantalate wafers, the use of a dry etching process tends to accumulate a charge and damage the fork fingers, and the wafer itself is also prone to damage. Compared with dry etching, the lift-off process has the advantages of having a small impact on the wafer, and it is a simple process; by precisely controlling the development time, a more uniform IDT can be obtained.

**Figure 7:**
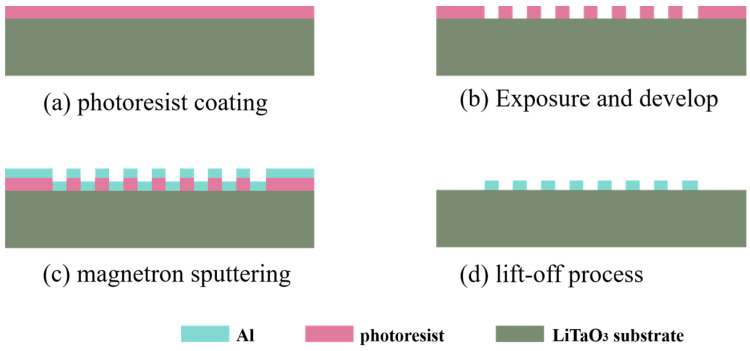
Device processing process flow chart.

Firstly, the organic and inorganic materials on the wafer substrate were cleaned off in different cleaning steps and then blown dry with a nitrogen gun. Then, the entire wafer was coated with photoresist spin using a spin coater; the type of photoresist was 6130, and the spin coating speed was 3000 r/min, followed by the exposure and development process. The photomask was used for the exposure process, in which the electrode weighting pattern was realized after using the MATLAB input program. Then, the Ti/Al (10/200 nm) film was used as the electrode of the SAW sensor using a magnetron sputtering system, and finally, the complete electrode was lifted off using an acetone solution. The titanium film was used as an adhesion layer to improve the adhesion ability of the aluminum film, and there was no significant effect on the device’s performance. The device processing flow chart is shown in Figure 7.

**Figure 8:**
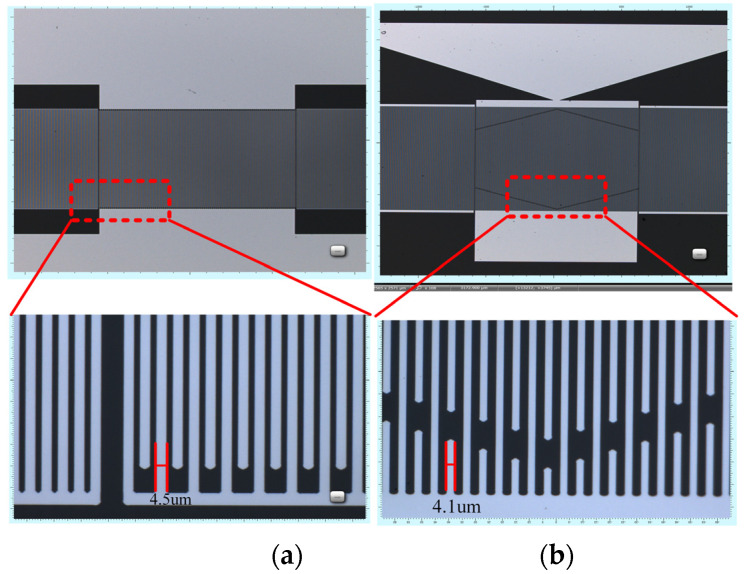
(**a**) Confocal microscope image of the unweighted resonator (Chip A). (**b**) Confocal micro scope image of the hexagonal weighted resonator (Chip B).

Both resonator base parameters were set at an IDT of 90 pairs and an electrode thickness of 200 nm, with the number of reflecting grids referencing the design scheme in the literature [26], which is designed to be 100 pairs and an aperture length set to 80 λ. These parameters were in accordance with the simulation in Table 1. Figure 8a,b shows the images of “Chip A” and “Chip B” under a confocal microscope. The analysis of the images shows that: the electrode pattern was well-fabricated and no adhesion was seen; the fork fingers were uniform and there were no broken fingers; the weighted IDT length decreased toward both ends and showed a hexagonal shape; the IDT finger width of “Chip A” was 4.5 μm, and its metallization rate was 56.25%; and the IDT finger width of “Chip B” was 4.1 μm, and its metallization rate was 51.25%.

## 3. Test Results and Discussion

**Figure 9:**
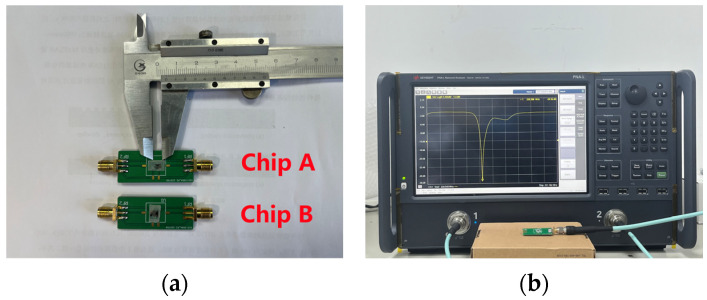
(**a**) Two types of devices made with test PCBs. (**b**) Test platform.

The device was tested by fixing the chip on a specific printed circuit board, using a lead bonding machine to connect the electrodes on the wafer to the PCB board with gold wires, as shown in Figure 9a. Before device testing, impedance matching of 50 Ω was performed. Then, it was connected to a microwave network analyzer (N5231B PNA-L, Keysight, Santa Rosa, CA, USA) using a coaxial line and characterized with S11 parameters using a standard single port, as shown in Figure 9b. The testing was conducted indoors at room temperature.

**Figure 10:**
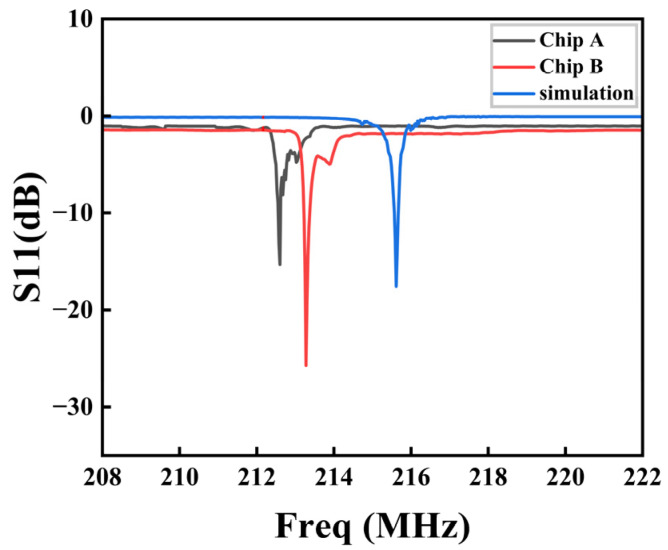
Comparison of actual measurement and simulation of chips A and B.

Chips A and B were tested under the same bonding and PCB conditions. Using a microwave network analyzer, “Chip A” and “Chip B” were measured, and their measured and simulated curves were compared, as shown in Figure 10. The simulation curve in Figure 10 is based on the simulation results in Figure 6c. Due to the PML structure at the bottom of the model absorbing a significant part of the bulk acoustic wave, the coupling between the spurious waves and bulk acoustic wave had decreased. As a result, the spurious waves on the right side of the main resonance were not visible in the simulation. The center frequencies of chips A and B were 212.7 MHz and 213.3 MHz, respectively, which slightly deviated from the simulation results of 215.8 MHz. The frequency deviation of chip A was greater than that of chip B. The reason for this may be that the IDT duty cycles of the completed resonator compared with that of the 50% design were different, and chip A’s difference was greater. It is also possible that the wafer’s cutting parameters were defective. In summary, the completed resonator test results were as expected. Repetitive testing was conducted on multiple resonators, and two representative test results were selected among them.

In this study, the quality factor (Q value) and the minimum return loss (RLmin) were used to characterize the resonator, which indicates the ability of the resonator to store energy and lose energy, respectively; these can reflect the performance of a resonator. The Q value was calculated using Formula (10), where $f_{r}$ represents the resonant frequency, and ($f_{1}-f_{2}$) represents the bandwidth size at −3 dB of the center frequency. The RLmin was calculated using the formula shown in Formula (11), where S11 is in a fractional form.

(10) $$Q\;value=\frac{f_{r}}{\left(f_{1}-f_{2}\right)}$$

(11) $${RL}_{min}=|\min\left\{20lgS11\right\}|$$

Because the weighted resonator can suppress the transverse spurious modes, it has a higher Q value and RLmin. The Q values of Chip A and Chip B were 10,910 and 10,406, respectively. The Q value of the resonator was slightly reduced by the weighting method, which follows the expression in this reference [27]. However, the Q value was still maintained at a higher level than that of the structure using the same substrate without weighting in the reference [28], indicating that the parameters designed in Table 1 are more reasonable.

**Figure 11:**
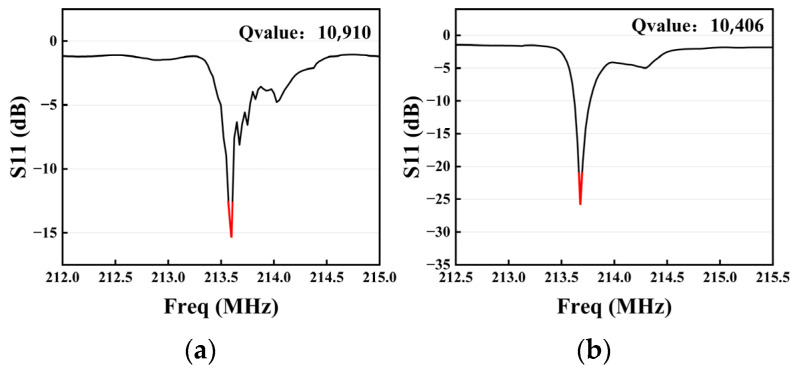
(**a**) Enlargement of actual measurement of device A. (**b**) Enlargement of actual measurement of device B.

Figure 11a shows the measured S11 amplification curve of Chip A. In the chart, the right side of the main resonance has the various orders of spurious responses, which have an overall serrated shape and a distribution range of up to 1 MHz. Spurious modes also largely affected the resonator’s ability to store energy, resulting in an RLmin value of only 15.7 dB, and the highest spurious peak accounted for 30% of the S11 curve for the main resonance. Although chip A had a high Q value, the presence of spurious mode also affected the RLmin. The S11 curve of chip B is shown in Figure 11b. In the same-sized resonant region, there was only a smooth curve of main resonance and a −4.8 dB side spurious peak, while the RLmin was up to 25.7 dB and the ratio of peak sidelobe was only 19%. Chip B reduced the ratio of the peak sidelobe, suppressing most of the spurious modes, while the Q value was not significantly reduced. The hexagonal weighted method improved the performance of the resonator to a large extent. Compared with the rhombic weighting scheme in reference [11], the hexagonal weighted resonator used in this study lost less of the aperture. Due to the increase in the number of IDTs and reflecting grids, the resonator can obtain a higher Q value and RLmin, which can better satisfy the use of surface acoustic wave devices.

In this study, the Q value and the RLmin of Chip A and Chip B were at a high level, and the RLmin of Chip B was better than that of Chip A due to the suppression of transverse clutter. In summary, the two structures of resonators designed in this study were reasonable and met the expected results.

## 4. Conclusions

In this study, a hexagonal weighted resonator structure with a spurious mode suppression function was proposed and designed, which could eliminate the spurious modes from adversely affecting the resonator. In this work, the simulation models were constructed to guide the parameter design of the resonator. The electrode thickness, electrode pair of IDTs, and aperture width were optimized to reduce the mass-loading effect, increase the admittance value, eliminate the effect of the sidelobe peak on the RLmin, and account for the excitation intensity of the resonator. The rational hexagonal weighting of the IDT eliminated the spurious modes that are prevalent in conventional resonators. Based on the simulation optimization results, two types of resonators were prepared using the MEMS process, in which the conventional resonator had a high Q value of 10,910 and a higher RLmin of 15.7 dB. Compared with the conventional resonator, the prepared hexagonal weighted resonator had a high Q value of 10,406, which also achieved a higher degree of spurious wave suppression, and the value of the RLmin and the ratio of peak sidelobe were improved by 64% and 11%, respectively. This work can be used as a reference for the design of high-performance SAW devices in the future.

## Figures and Tables

**Table 1:** Single-electrode unweighted resonator structure parameters.

Parameters	Value
Resonant frequency (f, MHz)	216
Wavelength (λ, μm)	16
IDT width (a, μm)	4
Al electrode thickness (h, nm)	200
Distance between IDTs and reflection grids (p, μm)	4
Aperture width	80 λ
Metallization rate	50%
the pair of IDTs	90

## Data Availability

Data are contained within the article.
